# Oral Delivery of a Novel Attenuated *Salmonella* Vaccine Expressing Influenza A Virus Proteins Protects Mice against H5N1 and H1N1 Viral Infection

**DOI:** 10.1371/journal.pone.0129276

**Published:** 2015-06-17

**Authors:** Zenglin Pei, Xiaohong Jiang, Zhu Yang, Xiaoguang Ren, Hao Gong, Michael Reeves, Jingxue Sheng, Yu Wang, Zishu Pan, Fenyong Liu, Jianguo Wu, Sangwei Lu

**Affiliations:** 1 State Key Laboratory of Virology, College of Life Sciences, Wuhan University, Wuhan, Hubei, China; 2 School of Public Health, University of California, Berkeley, California, United States of America; 3 Taizhou Institute of Virology, Taizhou, Jiangsu, China; 4 Jiangsu Affynigen Biotechnologies, Inc., Taizhou, Jiangsu, China; 5 Program in Comparative Biochemistry, University of California, Berkeley, California, United States of America; Lindsley F. Kimball Research Institute, UNITED STATES

## Abstract

Attenuated strains of invasive enteric bacteria, such as *Salmonella*, represent promising gene delivery agents for nucleic acid-based vaccines as they can be administrated orally. In this study, we constructed a novel attenuated strain of *Salmonella* for the delivery and expression of the hemagglutinin (HA) and neuraminidase (NA) of a highly pathogenic H5N1 influenza virus. We showed that the constructed *Salmonella* strain exhibited efficient gene transfer activity for HA and NA expression and little cytotoxicity and pathogenicity in mice. Using BALB/c mice as the model, we evaluated the immune responses and protection induced by the constructed *Salmonella*-based vaccine. Our study showed that the *Salmonella*-based vaccine induced significant production of anti-HA serum IgG and mucosal IgA, and of anti-HA interferon-γ producing T cells in orally vaccinated mice. Furthermore, mice orally vaccinated with the *Salmonella* vaccine expressing viral HA and NA proteins were completely protected from lethal challenge of highly pathogenic H5N1 as well as H1N1 influenza viruses while none of the animals treated with the *Salmonella* vaccine carrying the empty expression vector with no viral antigen expression was protected. These results suggest that the *Salmonella*-based vaccine elicits strong antigen-specific humoral and cellular immune responses and provides effective immune protection against multiple strains of influenza viruses. Furthermore, our study demonstrates the feasibility of developing novel attenuated *Salmonella* strains as new oral vaccine vectors against influenza viruses.

## Introduction

Influenza infection, which is caused by seasonal and pandemic influenza viruses, can potentially lead to large numbers of deaths in humans, animals, and domestic birds as well as significant financial losses [1]. For example, the World Organization for Animal Health highlighted the outbreaks of highly pathogenic avian influenza (HPAI) H5N1 virus, which is associated with a growing number of human zoonotic infections and has been met with intense public health interest [2, 3]. In 2009, a novel swine-origin H1N1 influenza A virus, which was initially identified in Mexico and spread globally, has continued to circulate in humans and may have the potential to develop into the first influenza pandemic of the twenty-first century [4–6]. To control and prevent potential outbreaks of influenza viruses (e.g. the H5N1 avian influenza virus and the 2009 swine-origin H1N1 virus) in the future, effective vaccines against influenza viruses are urgently needed.

Influenza viruses are members of the *Orthomyxoviridae* family, which have enveloped, segmented, single-stranded negative sense RNA genome [1]. The hemagglutinin (HA) is the most abundant viral membrane protein in the envelope and is responsible for both binding and fusion with host cells. The neuraminidase (NA), another viral membrane protein found in the virion, is pivotal in the release and spread of progeny virions, following the intracellular viral replication process [1]. It has been reported that influenza virus infections can be primarily and effectively controlled by vaccines that elicit both humoral and cellular immune responses against the viral surface proteins HA and NA [7–9]. Vaccines being used or explored against influenza viruses include conventional inactivated whole viral antigen vaccines, live attenuated reasserted virus vaccines, recombinant protein vaccines, virus-like particle (VLP) vaccines, and DNA vaccines [8, 10, 11]. DNA vaccine, as a novel vaccine candidate, has been shown to induce effective antibody response and long-term cell-mediated immunity in animal models [12–15]. Furthermore, DNA vaccines, when delivered orally, can induce systemic and mucosal immune as well as cellular immune responses to antigens as compared to vaccines delivered via the parenteral routes [9, 16]. These results suggest that orally delivered DNA vaccines may represent promising novel vaccines against influenza virus.

Oral vaccines are cost effective and operate conveniently because they eliminate the use of syringes and needles and thus are an affordable choice for mass vaccination. Attenuated *Salmonella* strains have successfully been used as an oral carrier system for delivery of nucleic acid-based vaccines [16, 17]. In previous studies, attenuated *Salmonella* were constructed and transformed with plasmid constructs containing transgenes under the control of an expression promoter [18–20]. In human cells infected by *Salmonella*, plasmid DNA can be released and transported to the nuclei, leading to the expression of the transgene [18, 21, 22]. The genes of *Salmonella* pathogenicity island 2 (SPI-2), which encode virulence factors and are required for intracellular survival and replication [23–25], are believed to play important roles in gene transfer ability of *Salmonella* vector. Inactivation of these genes, including the components of the type III secretion system (T3SS) encoded by SPI-2, led to better lysis of the bacteria and more efficient gene transfer [7, 18, 21, 26].

*Salmonella* may serve as promising oral vaccine vectors in vivo [19, 27]. Generation of new *Salmonella* strains with better gene transfer activity and further studies of these strains should facilitate the development of *Salmonella* as a vaccine vector against infectious pathogens including influenza viruses. In this study, we generated a novel attenuated *Salmonella* strain, SL368, with a deletion at the *spiR* gene, which is required for the expression of many SPI-2 genes [28]. Using SL368, we constructed a *Salmonella*-based vaccine expressing the HA and NA proteins of a human H5N1 influenza virus. We showed that SL368 exhibited efficient gene transfer activity and little virulence, leading to expression of viral antigens in cultured cells and in mice. Using BALB/c mice as the model, we studied the constructed *Salmonella*-based vaccine in comparison to the current commercial H5N1 and H1N1 influenza vaccines. Our study provides direct evidence that the SL368-based vaccine induces the production of anti-HA serum IgG and mucosal IgA, and of anti-HA interferon-γ producing T cells. Furthermore, mice orally vaccinated with the *Salmonella* vaccine were completely protected from lethal challenge of both highly pathogenic H5N1 and H1N1 influenza viruses. These results suggest that the *Salmonella*-based vaccine elicits strong antigen-specific humoral and cellular immune responses and provides effective immune protection against multiple strains of influenza viruses. Furthermore, our study demonstrates the feasibility of developing novel attenuated *Salmonella* strains as new oral vaccine vectors against influenza viruses.

## Materials and Methods

### Ethics Statement

This study was carried out in strict accordance with the recommendations in the Guide for the Care and Use of Laboratory Animals of the National Research Council (8th Edition). The animal experiment protocol was approved by the Animal Care and Use Committee of the State Key Laboratory of Virology, Wuhan University (Wuhan, China) or the Animal Care and Use Committee of the University of California-Berkeley (Protocol #R240). All efforts were made to minimize suffering of experimental animals.

### Virus strains, antibodies and synthetic peptides

Influenza virus A/Puerto Rico/8/34 (H1N1) and a reassortant H5N1 influenza virus (A/Viet Nam/1194R (H5N1)) were used in the study. The reassortant H5N1 virus (kindly provided by Dr. N. Robert (Virology Department, NIBSC, UK)) contains the HA and NA genes of human-isolated influenza virus (A/Viet Nam/1194/2004 (H5N1)) and the internal protein genes of A/Puerto Rico/8/1934 [15]. These viruses were propagated and grown in embryonated chicken eggs, following the procedure described previously [15, 29–31]. The anti-HA and anti-NA antibodies were purchased from GenScript (Piscataway, NJ) and the anti-actin antibody was from Sigma (St Louis, MO). The synthetic peptide IYSTVASSL, which represents amino acid residues 533–541 of the HA protein of the H5N1 influenza virus [32], was purchased from Sangon Biotech (Shanghai, China) and dissolved in water for use in subsequent assays. The commercial H5N1 and H1N1 vaccines, cv-H5N1 and cv-H1N1, which are inactivated vaccines being used for vaccination in humans in China, were purchased from Weike Inc (Harbin, China) and Hualan Inc (Henan, China), respectively.

### Construction of recombinant DNA plasmids

Constructs pMD-5HA and pMD-5NA contained the sequences coding for the HA and NA gene of the avian influenza virus A/Viet Nam/1194/2004 (H5N1) strain, respectively [15]. The H5N1 HA gene fragment was amplified from construct pMD-5HA by PCR using the primers 5HA-sense (5’- GGCAAGCTTCTGTCAAAATGGAGAAAAT-3’) and 5HA-antisense (5’- CGCTCGACTTTAACTACAATCTGAACT-3’). The PCR products were digested using Hind III and Xho I and then cloned into eukaryotic expression vector pVAX1 (Invitrogen, Carlsbad, CA, USA) to generate p5HA. Similarly, the H5N1 NA gene fragment was amplified from construct pMD-5NA by PCR using the primers 5NA-sense (5’- GTCAAGCTTACCATGAATCCAAATCAG-3’) and 5NA-antisense (5’- AGCTCGACCTACTTGTCAATGGTGAAT-3’), and then cloned into pVAX1 to generate p5NA.

### Construction and growth analysis of *Salmonella* strains

*Salmonella typhimurium* clinical strain ST14028s has been described previously [18, 33]. *Salmonella* strain SL368 was derived from the auxotrophic *Salmonella typhimurium aroA* strain SL7207 (a gift from Bruce A. D. Stocker, Stanford University, CA)[34] by deleting a part of the SpiR coding sequence with the λ Red recombinase method [35], following the procedures described in detail previously [33, 36]. Growth analysis of bacteria in LB broth was carried out with the experimental protocols as described in detail previously [33, 36].

### Expression of viral antigens by *Salmonella*-mediated delivery in cultured cells

*Salmonella* carrying different plasmid constructs, Sal-HA-NA and Sal-vector, were generated by transforming strain SL368 with p5HA and p5NA, or pVAX1, respectively. In gene transfer experiments, we infected HeLa cells and differentiated THP (1x10^6^ cells) (pretreated with IFN-γ (150 U/ml) (R&D Systems Inc., Minneapolis, MN) for at least 12 hours) with *Salmonella* at a multiplicity of infection (MOI) of 10–20 bacteria/cell [18, 21, 33]. The infected cultures were then incubated at 37°C in 5% CO_2_ for 1 hour. Cells were then washed five times with phosphate-buffered saline (PBS), and then incubated with fresh medium containing 50 μg/ml of gentamicin [18, 21, 33]. Cells were harvested after 72 hours incubation.

To assay the expression of the viral mRNAs, total RNAs were isolated from cells using Trizol (Invitrogen, San Diego, CA) and digested with DNase I to remove the genomic DNA. Northern and Western blot analyses were performed as described in detail previously [18, 21]. In Western blot analyses, we stained electrophoretically separated proteins using antibodies against influenza proteins and actin, and analyzed protein expression with a STORM840 Phosphorimager [18, 21].

### Oral immunization of mice

Specific-pathogen-free female BALB/c mice (6 weeks old) were obtained from the Laboratory Animal Centre, Yangzhou University (China) and housed in a specific pathogen free facility. Mice were randomly divided into groups (5–10 mice per group) and were immunization on day 0, 14, and 28. In experiments with *Salmonella*-based vaccines, mice were first anesthetized with isoflurane and then intragastrically inoculated with 0.1–0.2 mL phosphate-buffered saline (PBS) containing no *Salmonella* or 1 × 10^9^ cfu Sal-HA-NA or Sal-vector, using a gavage needle [18, 21, 37]. In experiments with the commercial H5N1 and H1N1 vaccines, mice were intramuscularly inoculated with cv-H5N1 (Weike Inc, Harbin, China) and cv-H1N1 (Hualan Inc, Henan, China) respectively, following the manufacturers’ recommendations. We have closely monitored the mice after the treatment. Even though no apparent side effects have been observed in mice vaccinated with the commercial influenza vaccines and SL368 carrying different plasmid constructs, we still took special precautions to ensure that ample food and water as well as sanitary cage conditions were present in order to maximize the animals’ comfort.

### Enzyme-linked immunosorbent assay (ELISA)

Sera samples were drawn from mice at indicated time points after immunization and before challenge with the viruses. Blood was spun at 13,000 rpm for 15 min, and the supernatant (serum) was removed and stored at -80°C. The levels of serum IgG and mucosal IgA-specific antibodies against influenza antigens were determined by ELISA with the mouse hemagglutinin HA IgG ELISA Kit and the mouse hemagglutinin HA IgA ELISA Kit (Xiangsheng Inc, Shanghai, China) respectively, following the manufacturers’ recommendations [7, 15]. Briefly, samples of test serum or mucosal nasal wash were added to triplicate wells, then mixed with diluted horseradish-peroxidase (HRP)-labeled anti-mouse IgG or anti-mouse IgA antibody (50 μl/well), and incubated at 37°C for 1 hour. Following several washes, the plates were incubated with 100 μl visualization reagent A and B of the HA IgG and IgA ELISA kits (Xiangsheng Inc, Shanghai, China) for 15 min. The reactions were stopped with 50 μl stop buffer and the absorbance was measured at 450 nm using an ELISA plate reader. The assays were performed in triplicate and the experiments were repeated three times. The values obtained were the average from these experiments.

### Hemagglutination inhibition (HI) assay

The serum neutralization activity was measured by HI assays [38]. Briefly, blood samples were collected at 0, 16, 32, 42 day after immunization, and specific receptor-destroying enzyme (RDE)-treated sera were serially diluted (2-fold) in V-bottomed 96-well plates, and then mixed with ~4 HA units of inactive influenza A/Puerto Rico/8/34(H1N1) or A/chicken/China/1204/04(H5N1). After incubating at room temperature for 30 min, 50 μl of 1% chicken erythrocytes was added to each well. The plate was incubated at room temperature for 40 min. The HI titer was defined as the highest serum dilution that showed 100% hemagglutination inhibition. The assays were performed in triplicate and the experiments were repeated three times. The values obtained were the average from these experiments.

### IFN-γ ELISPOT assay

The relative numbers of IFN-γ expressing T cells in single cell spleen suspensions were measured using the mouse IFN-γ ELISPOT kit (U-Cytech biosciences, Utrecht, Netherlands) as described previously [15]. Briefly, splenocytes (1×10^6^) were isolated from mice following euthanasia with overdose inhalational CO_2_, added to each well of pre-coated 96 well plate in triplicate, and stimulated with or without 100 μl/well (5 μg/ml) of HA_533–541_ epitope peptide (IYSTVASSL) [32] at 37°C for 24 hours. Phytohemagglutinin (PHA, 4 μg/ml, Sigma–Aldrich, St. Louis, MO, USA) was used as a positive control. After incubation, the cells were removed and incubated with 100 μl/well (0.5 μg/ml) of biotinylated detector antibody (anti-mouse IFN-γ) and 50 μl/well of 2% gold particle-labeled anti-biotin antibody (GABA) at 37°C for 1 hour. Finally, the plate was treated with Activator I/II at 37°C for 30 min. The number of spots was counted using a computer-assisted video image analyzer. The results were expressed as spot-forming cells (SFC) per million cells. The assays were performed in triplicate and the experiments were repeated three times. The values obtained were the average from these experiments.

### Studies of immunized mice challenged with influenza viruses

For viral challenge experiments, five mice per group were first anesthetized with isoflurane and then infected intranasally with 50 μl PBS (25 μl per nostril) containing a dose of 20 LD_50_ of lethal influenza virus strain A/Puerto Rico/8/34(H1N1) or A/Viet Nam/1194R (H5N1) two weeks after the final vaccination. The infected mice experienced the typical effects of systemic infection caused by influenza virus. We have taken special precautions and followed standard guidelines outlined in the Guide for the Care and Use of Laboratory Animals to ensure that ample food and water as well as sanitary cage conditions were present in order to maximize the animal’s comfort. Mice were observed daily to monitor body weight for 16 days. Humane (non-lethal) endpoints were used during the survival experiments. Animals were deemed gravely ill and were euthanized by overdose inhalation of carbon dioxide if they lost more than 30% of their weight or exhibited lethargy, ruffled hair coat, or hunched posture. Experiments suggested that inoculation of mice with a virus dose of 20 MLD50 is sufficient for our study as the viruses used are highly pathogenic. All efforts were made to minimize suffering of animals.

For histological analyses, mice were euthanized with overdose inhalational CO_2_. Lung tissues were collected and fixed with 10% formaldehyde, embedded in paraffin, and cut into 5 μm sections. Sections were stained with hematoxylin and eosin (H&E), and were then examined microscopically.

For determination of viral titers, lungs were isolated from mice at day 3 and 6 after virus inoculation. Lung tissues of equal weight were homogenized in DMEM medium to achieve 10-fold serially diluted suspensions of tissue homogenates and were titrated in 96-well culture plates of Madin-Darbin canine kidney (MDCK) cells. The titers were calculated by use of the Reed-Muench method and were expressed as log_10_TCID_50_/g lung tissue. The assays were performed in triplicate and the experiments were repeated three times. The values obtained were the average from these experiments.

### Statistical analyses

Statistical analyses of the data were performed using the analysis of variance (ANOVA). A p-value of less than 0.05 was considered significant.

## Results

### Construction of attenuated *Salmonella* vaccine

We have previously shown that attenuated *Salmonella* strains can be used as gene delivery vectors for expression of ribozymes in both human cells and in mice [18, 21, 39]. In this study, we constructed an attenuated *Salmonella* strain, SL368, as the gene delivery vector for the generation of the influenza vaccines. SL368 was derived from *Salmonella typhimurium* strain SL7207 [34] and in addition, contained a deletion of a part of the coding sequence of the SpiR protein. SL7207 is an attenuated strain with gene delivery activity [27, 34, 39, 40]. The SpiR protein is a part of the two-component transcription complex regulating the expression of the majority of the SPI-2 encoded genes, which are important for *Salmonella* intracellular survival and virulence in vivo [28, 41]. To generate *Salmonella* vaccine expressing influenza virus antigens, SL368 was electroporated with plasmid constructs p5HA and p5NA, which contained the DNA sequences coding for the HA and NA genes of a highly pathogenic influenza A H5N1 virus strain that were under control of a eukaryotic expression promoter, respectively (S1 Table).

Four vaccines were included in our study (S2 Table). Vaccine Sal-HA-NA contained strain SL368 carrying plasmid constructs p5HA and p5NA. Vaccine Sal-vector contained SL368 carrying the empty vector construct pVAX1 without any influenza virus sequences and was used as a negative control. We also included the commercial H1N1 and H5N1 vaccines, cv-H1N1 and cv-H5N1, as the positive controls in our study.

### Gene delivery and expression of influenza antigens in cultured cells and in mice mediated by attenuated *Salmonella*

**Fig 1:**
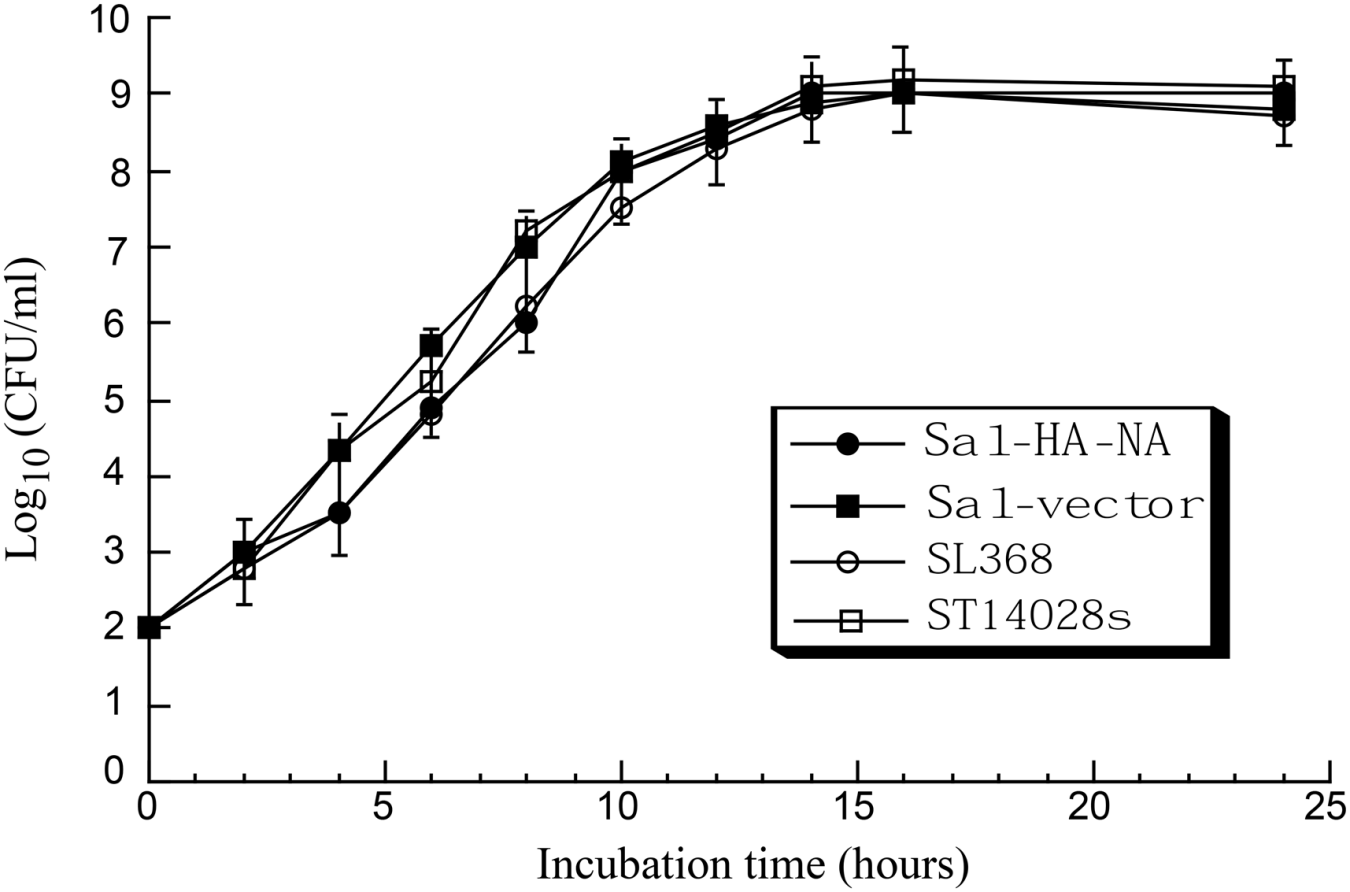
Analysis of growth in LB broth of *Salmonella*. *Salmonella* used in the study include the wild type strain ST14028s, mutant strain SL368, and its derivatives that carry the empty vector construct pVAX1 (Sal-vector), and constructs p5HA and p5NA (Sal-HA-NA). Experimental details can be found in Materials and Methods.

In growth analysis experiments, *Salmonella* carrying constructs containing expression cassettes for influenza virus genes grew in LB broth as well as bacteria carrying no constructs or carrying the pVAX1 empty vector (Fig 1). These results indicate that the presence of the influenza virus sequence does not result in an impaired viability of the bacterial carrier.

In Northern blot analyses, none of the influenza virus RNA transcripts was detected in *Salmonella* carrying constructs with viral sequences (data not shown). Furthermore, Western blot analyses using anti-influenza antibodies showed that neither influenza HA nor NA protein was detected in *Salmonella* grown in LB (data not shown). These results suggested that the viral proteins, which were under the control of a eukaryotic expression cassette, were not expressed when *Salmonella* grew in culture media outside of human cells.

**Fig 2:**
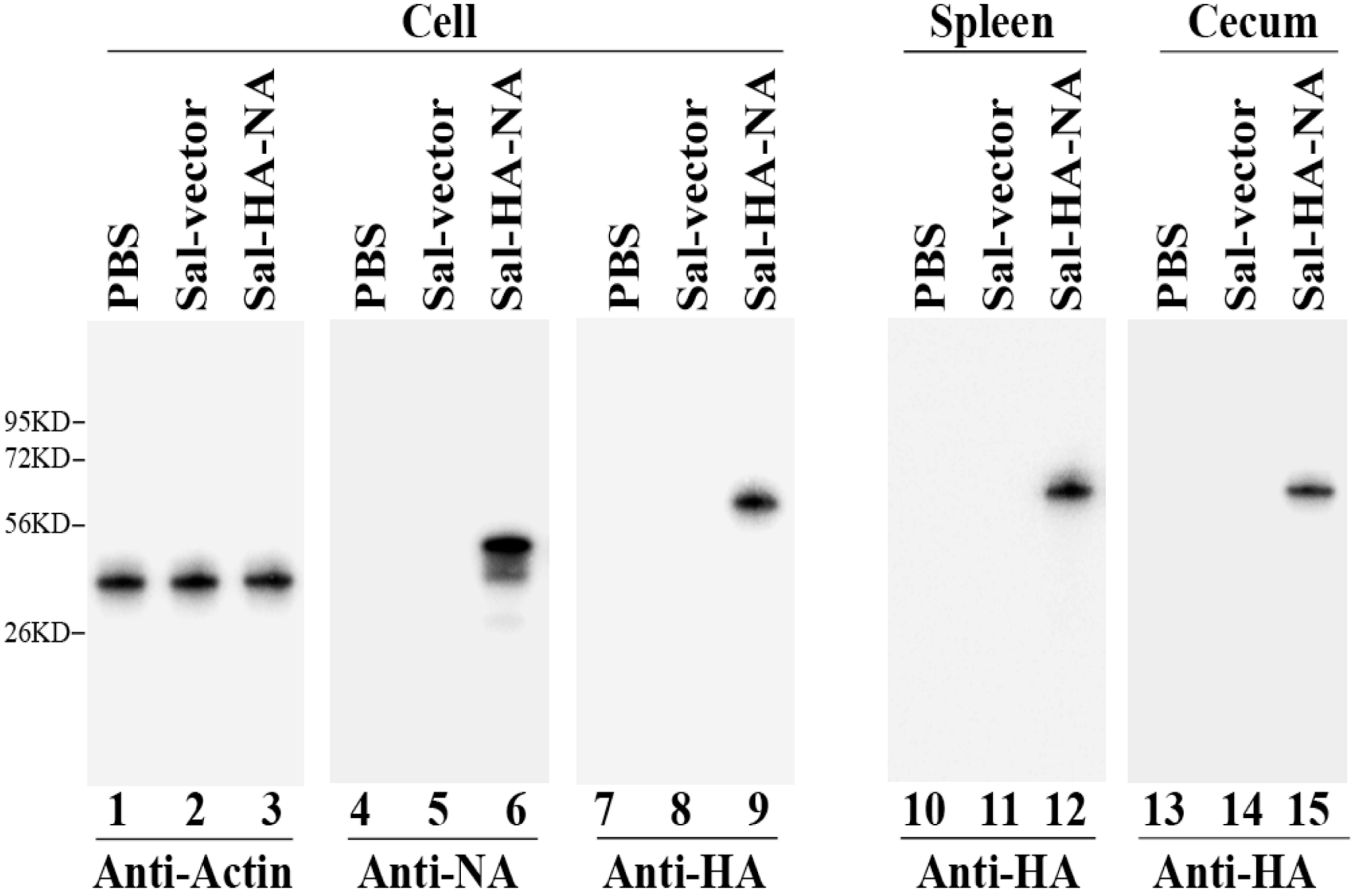
*Salmonella*-mediated delivery for influenza antigen expression in cells and in mice. Western blot analyses were performed to study the expression of viral HA and NA proteins in differentiated THP-1 cells (lanes 1–9) and viral HA protein in BALB/C mice (lanes 10–15) treated with phosphate-buffered saline (PBS) only (PBS, lanes 1, 4, 7, 10, and 13), SL368 carrying the empty vector construct pVAX1 (Sal-vector, lane 2, 5, 8, 11, and 14) and constructs p5HA and p5NA (Sal-HA-NA, lanes 3, 6, 9, 12, and 15). Cells treated with PBS or *Salmonella* were harvested at 72 hours post treatment while spleens and cecums were harvested from BALB/C mice that were intragastrically inoculated with PBS or SL368 carrying different constructs at 4 days after inoculation. Protein samples (40 μg) were separated on denaturing gels, transferred to membranes, and stained with antibodies against viral HA (lanes 7–15) and NA (lanes 4–6) or actin (lanes 1–3), which serves as the loading control. Molecular markers are shown on the left.

To determine whether *Salmonella* can efficiently deliver the influenza virus sequences into human cells, HeLa cells and differentiated macrophage THP-1 cells were infected with *Salmonella* SL368 carrying p5HA, p5NA, and the pVAX1 empty vector, and harvested at 72 hours postinfection. The expression of influenza HA and NA proteins was assayed with antibodies specifically against influenza virus proteins, using actin as the loading control (Fig 2). Western bolt analyses showed that *Salmonella* Sal-vector, in which SL368 carried the empty vector pVAX1, did not express any influenza antigen (Fig 2, lanes 5 and 8). The HA (~64KD) and NA (~50 KD) proteins were detected in cells infected with *Salmonella* Sal-HA-NA, in which SL368 carried constructs p5HA and p5NA (lanes 6 and 9). These results indicate that SL368 efficiently carried out gene transfer for delivery of constructs containing DNAs coding for viral antigens and that the HA and NA antigens were efficiently expressed following gene transfer of SL368 in human cells.

**Fig 3:**
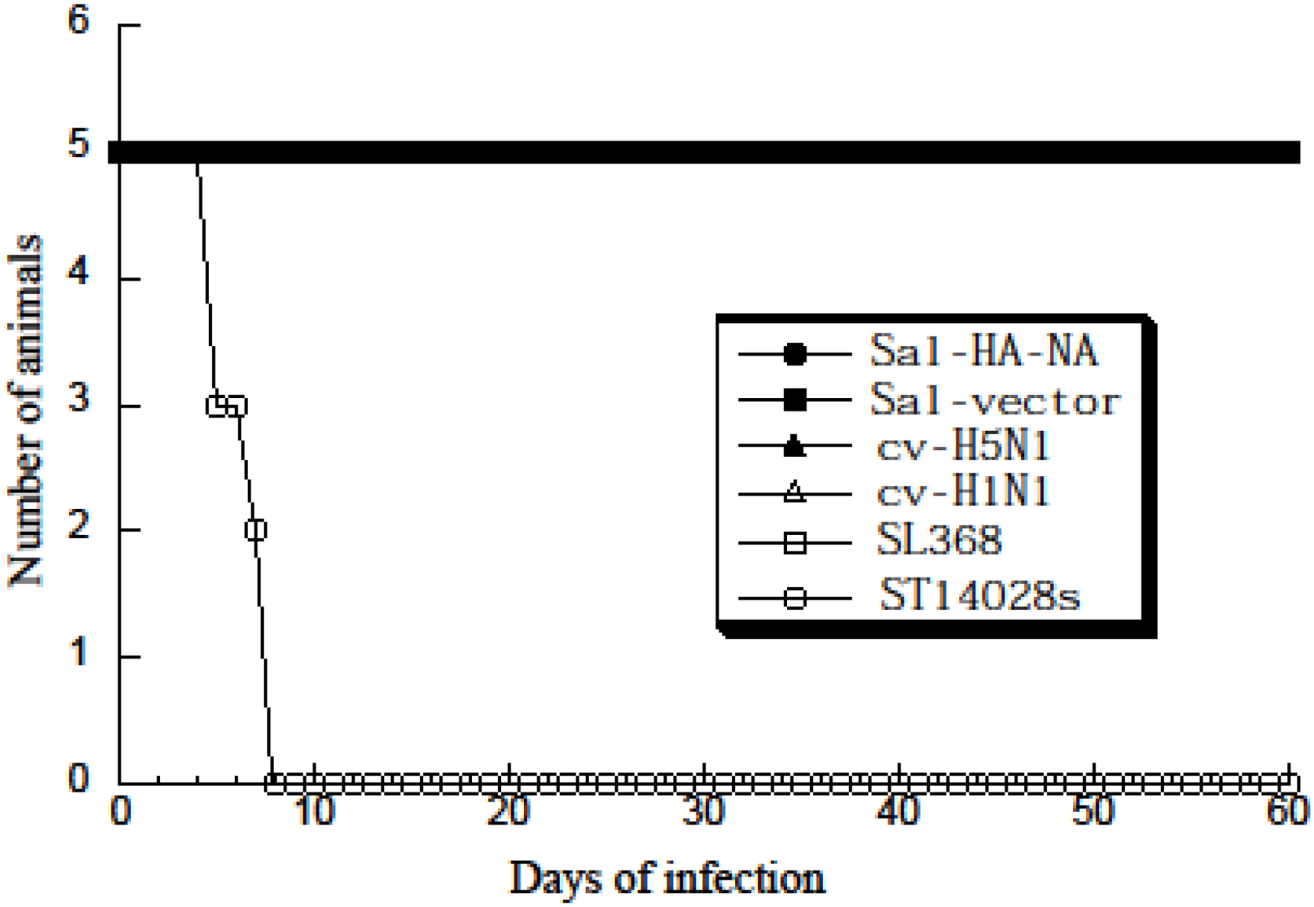
Toxicity and virulence of different *Salmonella* strains in BALB/c mice. Mice (5 animals per group) were either inoculated intramuscularly with the commercial H5N1 (cv-H5N1) and H1N1 vaccines (cv-H1N1) or intragastrically with PBS, the wild-type strain ST14028 (1 × 10^3^ CFU), and vaccine strain SL368 (1 × 10^9^ CFU) carrying the empty vector construct pVAX1 (Sal-vector) and constructs p5HA and p5NA (Sal-HA-NA). The mortality of the animals was monitored for 60 days. Humane (non-lethal) endpoints were used during the experiments and mice were euthanized with overdose inhalational CO_2_ to limit suffering, when they exhibited weight loss of more than 30%, lethargy, ruffled hair coat, or hunched posture.

To study *Salmonella*-mediated gene delivery for the expression of influenza virus antigens in vivo, we intragastrically inoculated BALB/c mice with *Salmonella* Sal-HA-NA and Sal-vector. Gene delivery mediated by Sal-HA-NA was efficient in vivo as HA and NA proteins were detected in the intestines and spleens of the *Salmonella*-treated mice (Fig 2, lanes 10–15, data not shown). Furthermore, SL368 carrying no construct, the viral antigen constructs, and the empty vector pVAX1 exhibited much less virulence in vivo than the wild-type strain ST14028s. All mice infected with SL368, Sal-HA-NA, and Sal-vector (1 × 10^9^ cfu/mouse) remained alive even at 60 days postinoculation (Fig 3). In contrast, mice inoculated with a much lower dose of ST14028s (1 × 10^3^ cfu/mouse) died within 7 days (Fig 3). Thus, the constructed SL368-based vaccine appeared to be efficient in gene transfer and exhibited little virulence and pathogenicity in vivo.

### Humoral responses induced by the vaccines

BALB/c mice were randomly divided into groups and vaccinated intragastrically with the constructed *Salmonella* Sal-HA-NA and Sal-vector at day 0, 14, and 28. Animals were also treated similarly with phosphate-buffered saline (PBS) in the absence of *Salmonella* and used as the negative controls. To compare the effectiveness of our generated *Salmonella*-based vaccines, vaccines cv-H5N1 and cv-H1N1, which were currently commercially available for vaccination in China against H5N1 and H1N1 viral infections respectively, were used as positive controls and injected intramuscularly into mice at day 0, 14, and 28. All animals remained alive at day 42 post immunization (i.e. two weeks after the final immunization) (data not shown), consistent with our observations (Fig 3) that SL368 exhibited little virulence/pathogenicity in mice in vivo.

**Fig 4:**
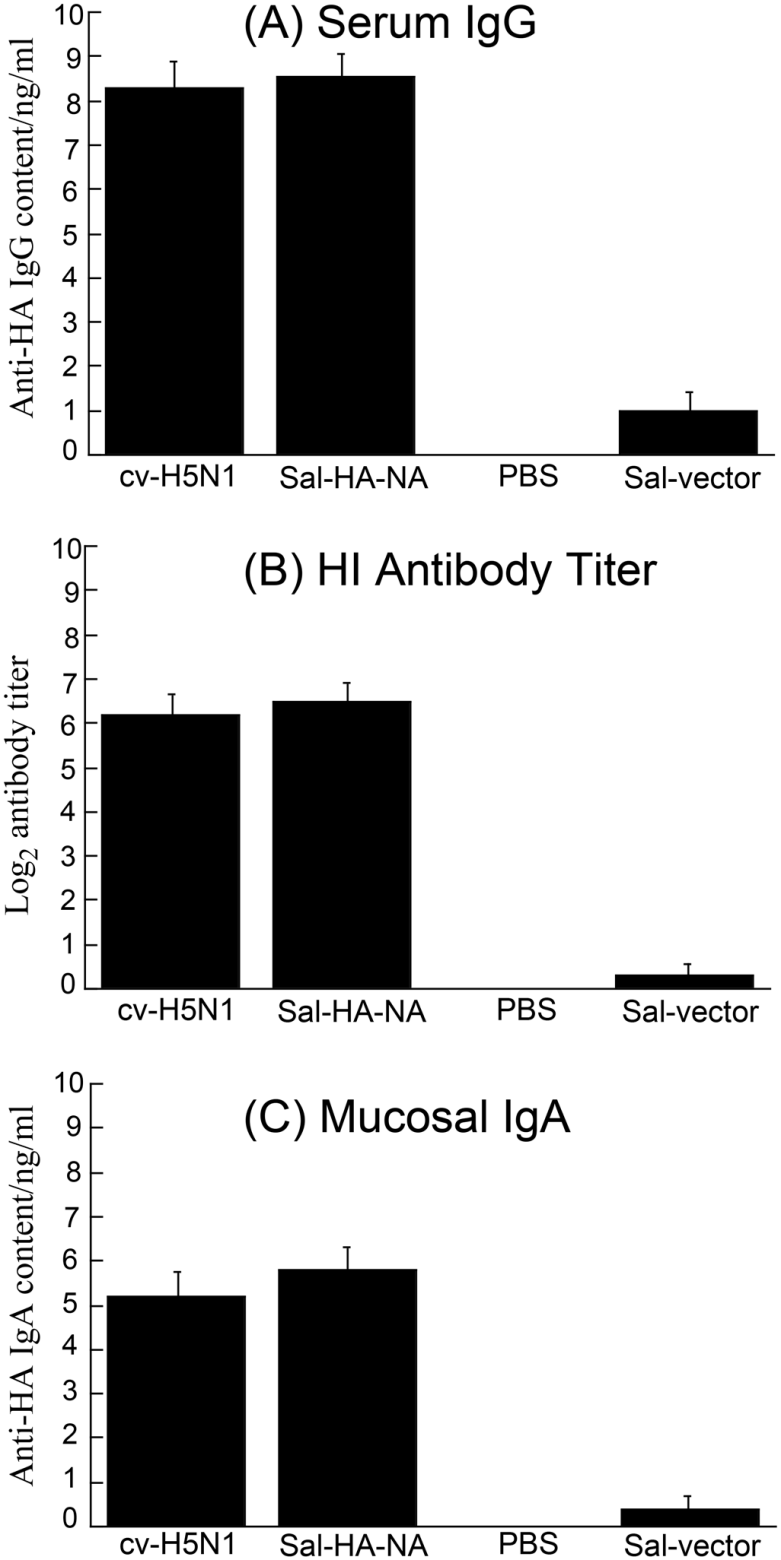
Antibody titers of anti-HA serum IgG (A), serum hemagglutination inhibition (HI)(B), and anti-HA mucosal IgA (C) detected by ELISA in immunized mice at 42 days after immunization. Mice were immunized intragastrically at day 0, 14, and 28 with PBS only, the commercial H5N1 vaccine (cv-H5N1), and *Salmonella* SL368 carrying the empty vector pVAX1 (Sal-vector) and constructs p5HA and p5NA (Sal-HA-NA). Pool serum or mucosal wash samples (n = 5) from mice within a group were assayed and analyzed by two-way ANOVA. *p < *0*.*01*. The assays were performed in triplicate and the experiments were repeated three times. The values obtained were the average from these experiments.

To study the effects of *Salmonella* vaccines on humoral responses in BALB/c mice, ELISA was carried out to assay the levels of serum antibodies at 0, 16, 32, 42 day after immunization (Fig 4) [42]. At day 42, mice immunized with vaccine Sal-HA-NA, in which SL368 carried constructs p5HA and p5NA, displayed higher (*p* < *0*.*01*) anti-HA serum IgG levels compared to those immunized with the control vaccine Sal-vector, which carried the empty vector pVAX1 (Fig 4A). Indeed, Sal-HA-NA induced similar anti-HA serum IgG levels as the H5N1 commercial vaccine cv-H5N1 (Fig 4A). The functional activities of the sera from the vaccinated mice were further investigated by determining the antibody titers for hemagglutination inhibition (HI) against influenza virus. Consistent with our results of the serum anti-HA IgG response induced by the vaccines (Fig 4A), mice immunized with Sal-HA-NA also displayed comparable HI antibody titers as those immunized with the commercial H5N1 vaccine, cv-H5N1, and exhibited higher HI antibody titers (*p* < *0*.*01*) than those with control Sal-vector (Fig 4B).

To study the effects of *Salmonella* vaccines on mucosal antibody responses in BALB/c mice, ELISA was also used to assay the anti-HA IgA levels in the mucosal nasal wash at 0, 16, 32, 42 days after immunization [43]. Mice immunized with vaccine Sal-HA-NA displayed higher anti-HA IgA levels (*p* < *0*.*01*) compared to those immunized with control Sal-vector (Fig 4C). Indeed, vaccine Sal-HA-NA induced similar levels of mucosal anti-HA IgA as the H5N1 commercial vaccine cv-H5N1 (Fig 4C). These results suggest that the SL368-based vaccine enhances antigen-specific mucosal IgA as well as serum IgG responses.

### Antigen specific T cell responses induced by the vaccines

**Fig 5:**
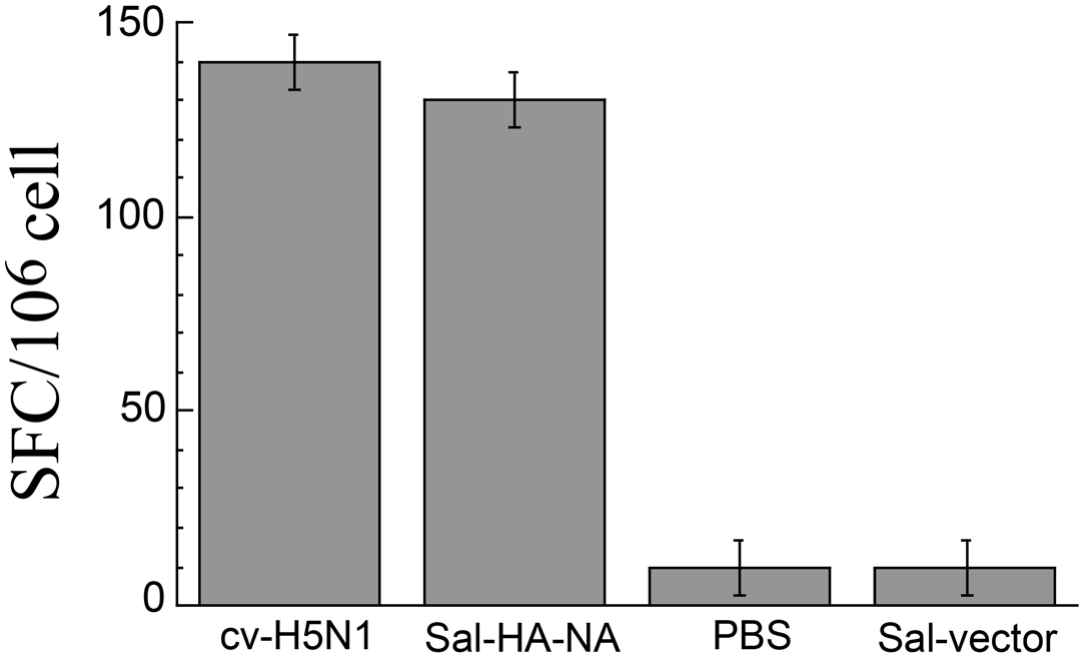
ELISPOT analysis of IFN-γ production by HA_533–541_ specific T cells in vaccinated mice at 42 days after immunization. Mice were immunized intragastrically at day 0, 14, and 28 with PBS, the commercial H5N1 vaccine (cv-H5N1), and *Salmonella* SL368 carrying the empty vector pVAX1 (Sal-vector) and constructs p5HA and p5NA (Sal-HA-NA). Splenocytes (n = 5) were harvested from immunized mice at 42 days post immunization and stimulated with HA_533–541_ peptide for 48 hours. The results were expressed as spot-forming cells (SFC) per million cells. The assays were performed in triplicate and the experiments were repeated three times. The values obtained were the average from these experiments. Statistical analysis was performed by two-way ANOVA. *p < *0*.*01*.

To study the HA-specific T cell responses induced by attenuated *Salmonella* vaccines, splenocytes (10^6^ cells) were isolated from vaccinated mice 42 days after oral administration, and stimulated with HA_533–541_ epitope peptide (IYSTVASSL). This peptide, which is common to most of influenza A virus strains, is considered to be an immunodominant MHC class I epitope (K^d^ restricted) and can induce CD8+ T-cell responses in BALB/C mice [32]. *Salmonella* Sal-HA-NA induced higher (*p* < *0*.*01*) anti-HA IFN-γ producing T cell responses than control Sal-vector (Fig 5). Indeed, the level of IFN-γ producing T cell responses induced by Sal-HA-NA was comparable to that by the commercial H5N1 vaccine cv-H5N1 (Fig 5). These results suggest that SL368-based vaccines induce antigen-specific T cell responses.

### Immune protection of mice from virus challenge induced by the vaccines

**Fig 6:**
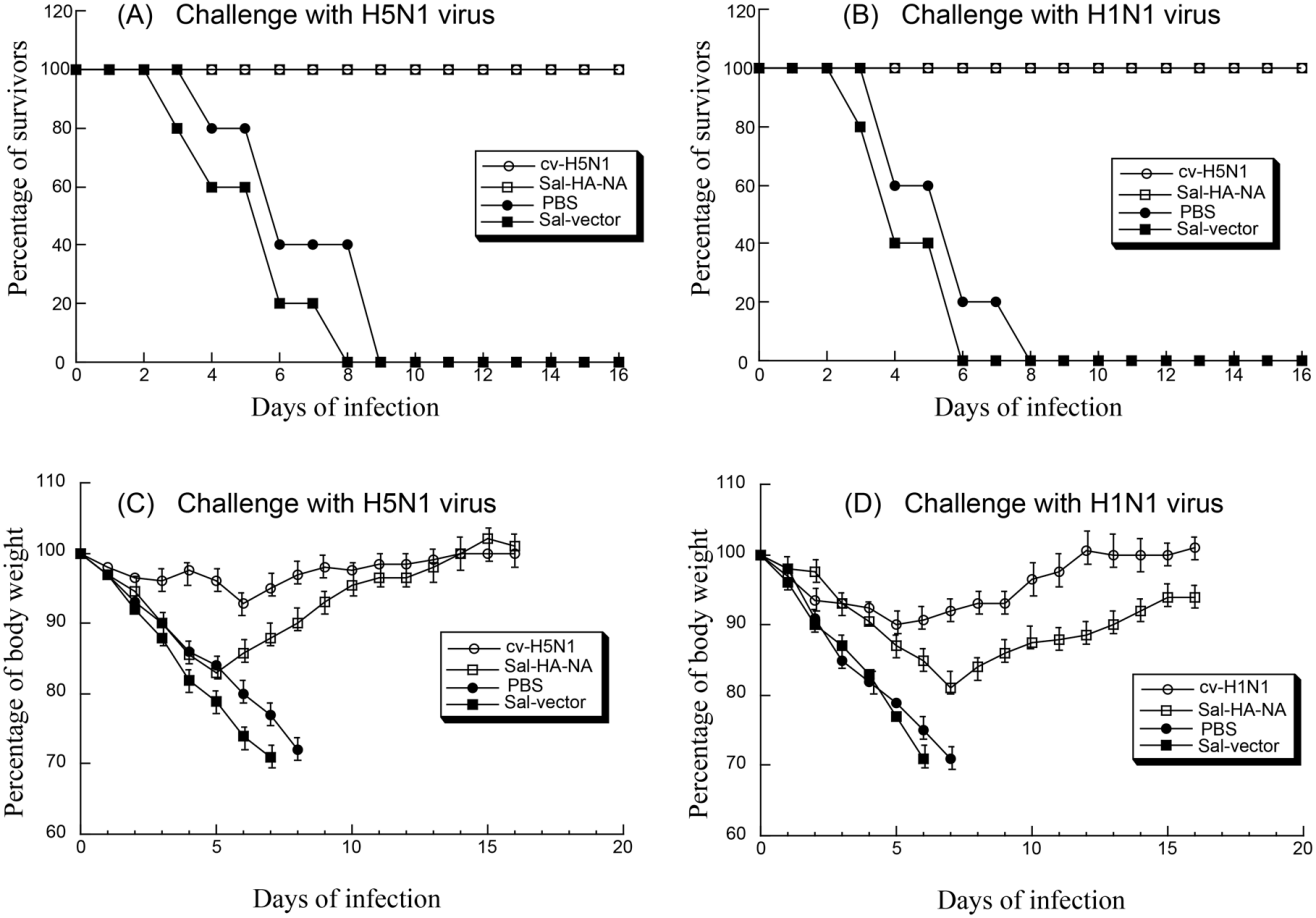
Immune protection of mice from lethal H5N1 (A and C) and H1N1 viral challenge (B and D). Groups of mice (n = 5) were intragastrically immunized three times at day 0, 14, and 28 with PBS only, the commercial H5N1 (cv-H5N1) and H1N1 vaccines (cv-H1N1), and *Salmonella* SL368 carrying the empty vector pVAX1 (Sal-vector) and constructs p5HA and p5NA (Sal-HA-NA), and then challenged intranasally with 20 LD_50_ of lethal H5N1 (A/Viet Nam/1194R) and H1N1 (A/Puerto Rico/8/34) viruses at two weeks after final immunization (i.e. at 42 days post initial immunization). Mice were monitored for survival (A and B) and weight loss (C and D) throughout a 16 day observation period. The results are presented in terms of percent survival and percent of body weight (at the beginning of the trial) respectively. Humane (non-lethal) endpoints were used during the survival experiments and mice were euthanized with overdose inhalational CO_2_ when they exhibited weight loss of more than 30%, lethargy, ruffled hair coat, or hunched posture.

Our observations of strong virus specific humoral and cellular responses suggest that vaccine Sal-HA-NA may provide immune protection to challenge of the H5N1 virus and, possibly, H1N1 virus since the NA proteins of both viruses are similar. To determine if the constructed *Salmonella* vaccines can induce H5N1 virus-specific and H1N1 virus-specific immune protection, mice were immunized with different vaccines at day 0, 14, and 28, and then isoflurane-anesthetized and intranasally challenged with lethal doses of highly pathogenic H1N1 and H5N1 viruses at day 42 (i.e. two weeks after the final immunization). Animals were closely monitored for weight loss and mortality for 16 days post challenge (Fig 6A–6D). Humane (non-lethal) endpoints were used during the survival experiments and mice were euthanized with overdose inhalational CO_2_ to limit suffering, when they exhibited weight loss of more than 30%, lethargy, ruffled hair coat, or hunched posture. Our results showed that mice vaccinated with the H5N1 commercial vaccine cv-H5N1 and vaccine Sal-HA-NA exhibited no loss of body weight and showed 100% protection against A/Viet Nam/1194R (H5N1) virus challenge after 16 days post challenge (Fig 6A and 6B). When challenged with A/Puerto Rico/8/34 (H1N1) virus, the mice vaccinated with the H1N1 commercial vaccine cv-H1N1 exhibited no body weight loss and showed 100% protection (Fig 6C and 6D). All mice immunized with Sal-HA-NA survived after challenge with A/Puerto Rico/8/34 (H1N1) virus and regained about 90% body weight by day 16 after challenge (Fig 6C and 6D). Mice administered with control Sal-vector or PBS exhibited significant body weight loss and exhibited no protection against either H5N1 or H1N1 virus challenge (Fig 6). These results suggest that SL368-based vaccine provides immune protection against both H5N1 and H1N1 influenza viruses.

**Fig 7:**
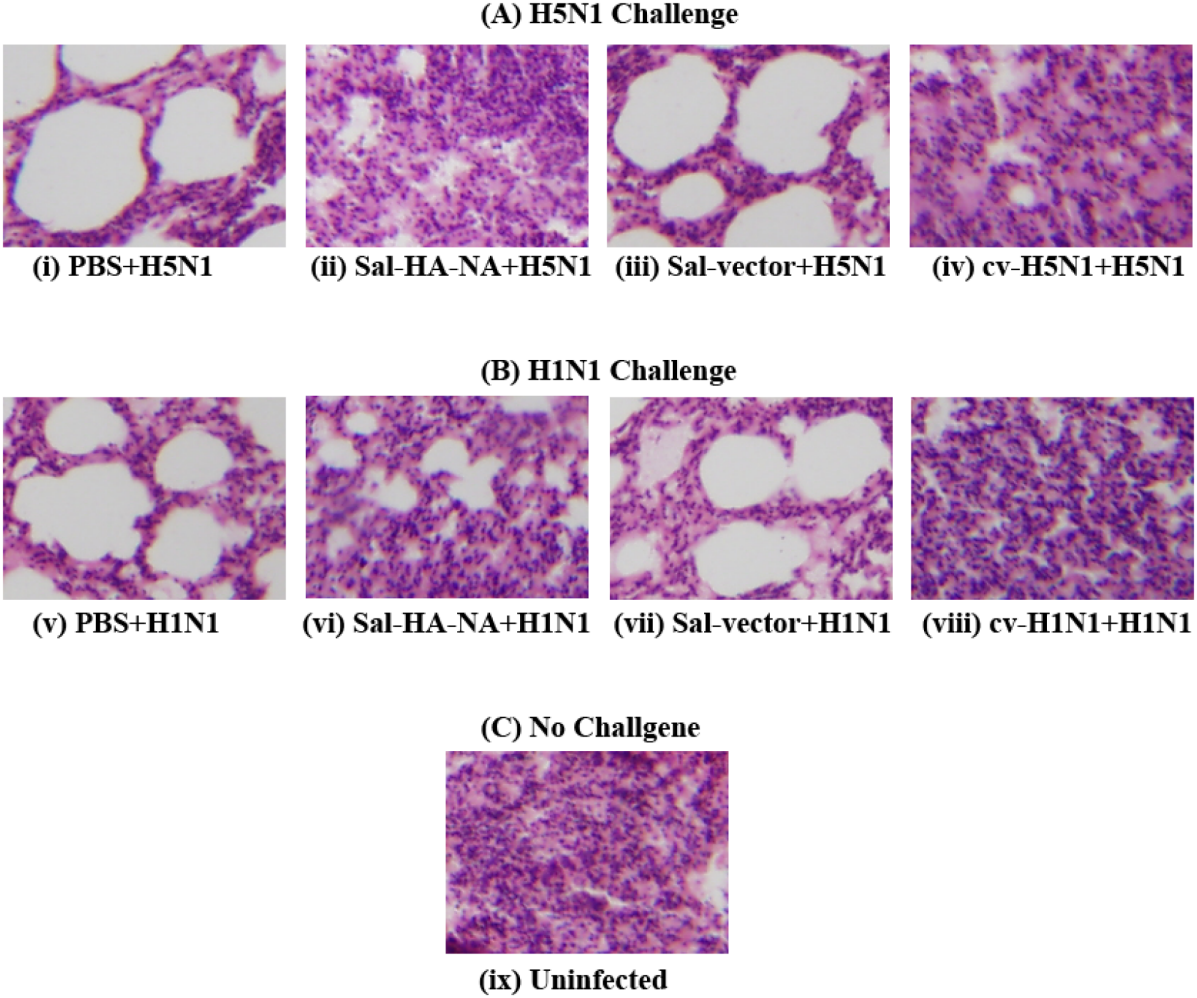
Photomicrographs of hematoxylin- and eosin-stained lung sections of mice at 7 days post-challenge. Mice were intragastrically immunized three times at day 0, 14, and 28 with PBS (i and v), the commercial H5N1 (cv-H5N1) (iv) and H1N1 vaccines (cv-H1N1) (viii), and *Salmonella* SL368 carrying the empty vector pVAX1 (Sal-vector) (iii and vii) and constructs p5HA and p5NA (Sal-HA-NA) (ii and vi), and then challenged intranasally with 20 LD_50_ of lethal H5N1 (A/Viet Nam/1194R) and H1N1 (A/Puerto Rico/8/34) viruses at two weeks after final immunization. (A) mice challenged with H5N1 virus; (B) mice challenged with H1N1 virus; (C) uninfected mice with no virus challenge.

**Fig 8:**
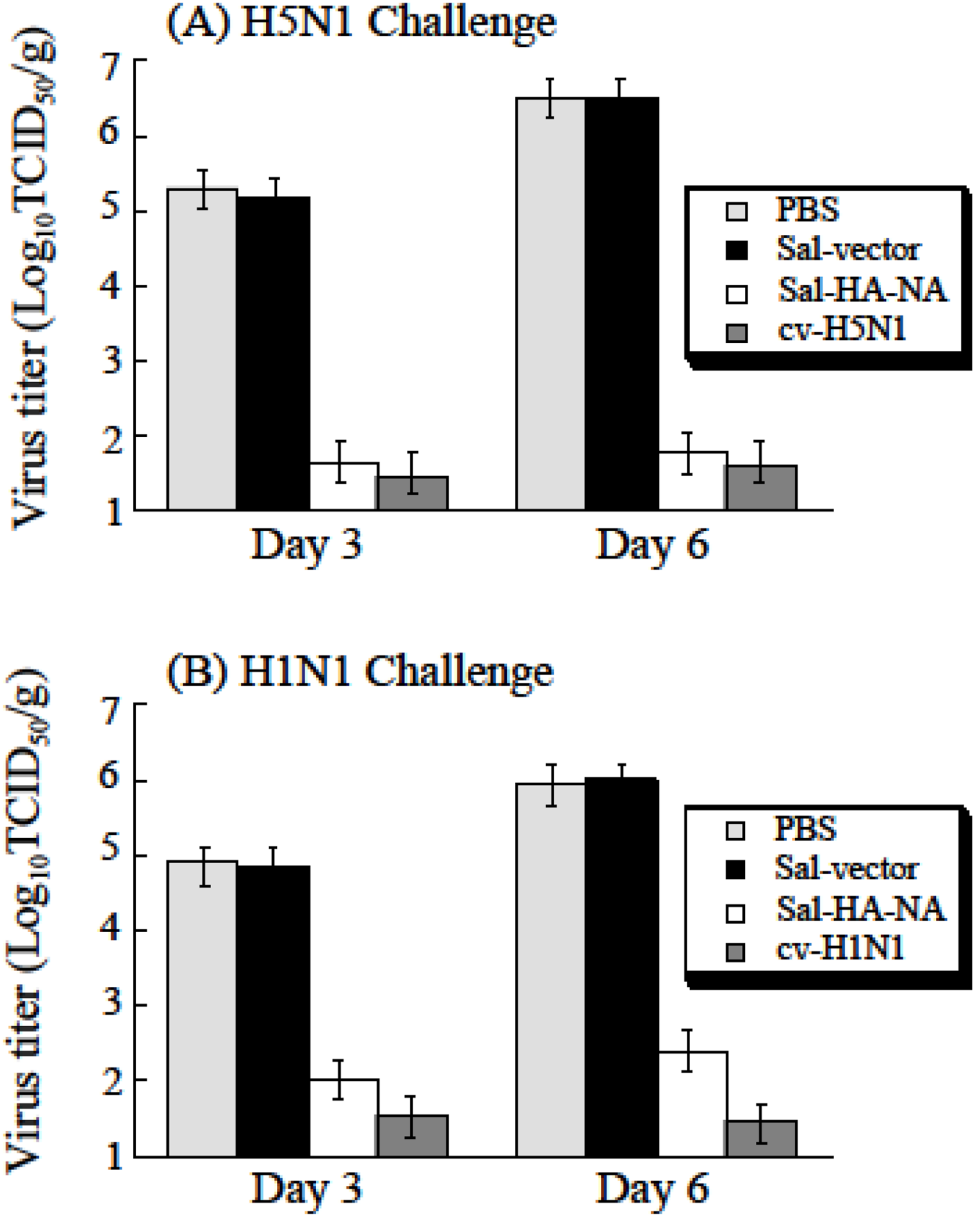
Virus titers in the lungs of mice challenged with H5N1 and H1N1 viruses. Mice were intragastrically immunized three times at day 0, 14, and 28 with PBS, the commercial H5N1 (cv-H5N1) and H1N1 vaccines (cv-H1N1), and *Salmonella* SL368 carrying the empty vector pVAX1 (Sal-vector) and constructs p5HA and p5NA (Sal-HA-NA), and then challenged intranasally with 20 LD_50_ of lethal H5N1 (A/Viet Nam/1194R) and H1N1 (A/Puerto Rico/8/34) viruses at two weeks after final immunization. Lung tissues were harvested at 3 and 6 days post challenge and viral titers were determined. The experiments were performed in triplicate and were repeated three times, and the standard deviation is indicated by the error bar. The limit of detection was 1.0 log_10_TCID_50_/g.

To further study the effect of the *Salmonella* vaccines on virus-specific immune protection, histological studies were performed with the mice vaccinated and challenged with A/Viet Nam/1194R (H5N1) and A/Puerto Rico/8/34 (H1N1) (Fig 7). At 7 days post-challenge, the lung of the animals were harvested immediately following euthanasia, fixed with 10% formaldehyde, embedded in paraffin and cut into sections. Sections were stained with hematoxylin and eosin, and then examined for histopathologic changes. As expected, no lesion was found in the lungs of uninfected mice (Fig 7C). Mice treated with PBS or control Sal-vector had pulmonary lesions consisting of substantial necrotizing bronchitis and histocytic alveolitis with associated pulmonary edema (Fig 7A(i, iii) and 7B(v, vii)). Mice vaccinated with vaccine Sal-HA-NA, and the H5N1 and H1N1 commercial vaccines (cv-H5N1 and cv-H1N1) lacked lesions in the lung and exhibited minimal bronchitis (Fig 7A(ii, iv) and 7B(vi, viii)). To investigate if immunization with Sal-HA-NA can inhibit local replication of the challenge viruses, lung tissues were harvested from animals at 3 and 6 days post-challenge and the virus titers in these tissues were determined. The titers of H5N1 virus in animals vaccinated with Sal-HA-NA and cv-H5N1 at 6 days post challenge were at least 10,000 fold lower than those in animals administered with PBS or Sal-vector (Fig 8A). Similarly, the titers of H1N1 virus in animals vaccinated with Sal-HA-NA and cv-H1N1 at 6 days post challenge were at least 2,000 fold lower than those in animals administered with PBS or Sal-vector (Fig 8B). These observations are consistent with our immune protection results (Fig 6) and suggest that the SL368-based vaccines can induce effective protection against highly pathogenic H5N1 and H1N1 influenza virus challenge.

## Discussion

Influenza A viruses continue to pose a public health threat to humans and animals [1]. Consequently, there is an urgent need for effective vaccines against these emerging infectious pathogens [44]. Intranasal influenza virus vaccines, while effective, may not be suitable for persons with asthma and certain chronic airway or pulmonary diseases [45]. Oral vaccines may represent an alternative to be used against influenza virus infection. In addition, oral vaccines are cost effective and operate conveniently because they eliminate the use of syringes and needles and thus are an affordable choice for mass vaccination. In this report, a novel attenuated *S*. *typhimurium* strain, SL368, was constructed and used as an oral vaccine vector for expressing HA and NA proteins of H5N1 influenza virus. Using BALB/c mice as the model system, we showed that the *Salmonella*-based vaccine, Sal-HA-NA, elicited anti-HA-specific humoral and T cell responses and induced immune protection against H5N1 and H1N1 virus challenge in immunized mice. These results provide direct evidence to suggest that attenuated *Salmonella* (*spiR* ^-^) strains expressing viral antigens represent promising oral vaccines against influenza virus infection.

Attenuated *Salmonella* represent unique and promising gene delivery tool for developing vaccine against infectious pathogens. First, as vaccines, the *Salmonella*-based vectors may be safe and can be administered orally. The currently-used antityphoid fever vaccine is derived from an attenuated *Salmonella* strain [46, 47]. Second, in the poultry industry, attenuated *Salmonella* vaccines might have unique potential for anti-influenza application, where inexpensive mass immunization can be achieved through drinking water or spray cabinet administration [48]. Finally, *Salmonella*-based vectors are highly immunogenic in inducing both innate and adaptive immune responses, due to the bacteria-encoded surface antigens such as lipopolysaccharide (LPS), and therefore can enhance the antigen-specific immune responses induced by the expression of the viral proteins [46, 49].

Attenuated *Salmonella* strains have been shown to function as oral vaccines against influenza virus infections by expressing different viral antigens [7, 26, 50]. For example, the recent seminal studies carried out by Curtiss and colleagues have shown the utility of attenuated *Salmonella* strains as oral vaccine candidates against influenza viruses [7, 26]. Anti-influenza vaccines were constructed based on an attenuated *Salmonella* mutant with the deletion of *sif*A gene [7]. The *sifA* gene encodes a secreted effector protein of the Type III secretion system (T3SS) of SPI-2 [51]. The SPI-2 proteins are important for *Salmonella* intracellular survival and virulence in vivo [23–25]. *Salmonella*-based vaccines with mutations in the *sifA* gene have been shown to elicit both humoral and cellular immune responses and induce immune protection against influenza virus infection in mice [7, 26].

In our study, attenuated *Salmonella* strain SL368 was derived from auxotrophic strain SL7207 [34] and, in addition, contained a deletion of a part of *spiR*. SpiR is required for the expression of many *Salmonella* Pathogenicity Island-2 (SPI-2) genes including SifA [28]. Thus, SL368, with a deletion at *spiR*, is expected to exhibit minimal virulence (Fig 3) and be even less virulent than those mutants with the deletion of a single SPI-2 T3SS factor such as *sifA*. Moreover, SL368 exhibited excellent gene transfer activity as efficient expression of viral HA and NA proteins was found in cells and in mice treated with the SL368-derived vaccines (Fig 2). Furthermore, the SL368-derived vaccines elicited both HA-specific humoral and T cell immune responses and induced immune protection against influenza virus infection in mice (Figs 4–8). These results suggest that attenuated *Salmonella* strains with the mutations at *spiR* represent novel vaccine vectors against influenza virus infection.

The exact mechanism of how *Salmonella* carry out gene transfer is not currently understood completely. In our delivery system, attenuated *Salmonella* were constructed and transformed with plasmid constructs containing transgenes (i.e. HA and NA) under the control of a eukaryotic expression promoter [18–20]. In cells (e.g. macrophages) infected by *Salmonella*, plasmid DNA can be released and transported to the nuclei, leading to the expression of the transgene [18, 21, 22]. As under the eukaryotic expression promoter, the antigens are only expressed and processed for antigen presentation in *Salmonella*-infected cells (e.g. macrophages and dendritic cells) and are not exposed to the environment of the digestive tract. Once inside the cells, the attenuated *Salmonella* will be lysed and the plasmid DNA will be released and transported to the nuclei. The antigen expression cassette should be in a plasmid instead of the bacterial genome to facilitate its transport to the nuclei. Additional studies on the process and mechanism of *Salmonella*-mediated gene delivery would facilitate the generation of *Salmonella*-based vectors for vaccine development.

One of the ultimate goals for prevention of influenza outbreak is to develop a vaccine that can provide cross-protective immunity against many influenza virus strains. It is interesting to note in our results that vaccine Sal-HA-NA, which expressed the HA and NA proteins of an H5N1 virus, also provided strong protection against an H1N1 virus strain in addition to the H5N1 virus. Our results of the cross-protective immunity by Sal-HA-NA suggest that attenuated *Salmonella* vector simultaneously carrying expression cassettes for various antigens can function as a vaccine candidate against multiple strains of influenza viruses.

Little is currently known about how our vaccine elicited cross-protective immunity. It is possible that the HA protein expressed in Sal-HA-NA, which is from an H5N1 virus, may elicit cross-protective immune responses against the H1N1 virus, because of potential HA sequence homology between H5N1 and H1N1 viruses [52]. Furthermore, it is also conceivable that the cross-protective immunity against multiple influenza viruses induced by Sal-HA-NA may be due to the expression of the NA protein, which is also from an H5N1 virus and which may share sequence homology with the NA proteins from H1N1 viruses. Previous studies have implicated the roles of NA in cross-protective immunity against influenza viruses [53, 54]. A VLP vaccine expressing the N1 protein was found to elicit cross-protective responses against H5N1 and H1N1 viruses while a DNA vaccine designed to generate anti-human N1 response could induce partial cross immunity against H5N1 virus [53, 54]. It will be interesting to determine if Sal-HA-NA vaccine may also elicit immunity against other influenza A viruses expressing HA and NA antigens such as H3N2 and H7N9 viruses. Further studies on the nature of immune responses elicited by Sal-HA-NA and other *Salmonella*-based vaccines against these viruses should provide insight into developing novel vaccines against influenza virus infections.

We recognize that the BALB/c mice model used in our study has limitations in the evaluation of the constructed vaccines. The immune responses observed in the mouse model may not truly reflect those in humans when immunized with the vaccines and infected with influenza viruses. Additional studies are needed to study the effect of *Salmonella*-based influenza vaccines in other animal models as well as in humans. Future studies on these issues, as well as on the construction of new attenuated *Salmonella* strains with novel mutations for expressing different viral proteins and antigenic regions, should facilitate the development of *Salmonella*-based vaccines against influenza virus infection.

## Supporting Information

S1 Table *Salmonella* strains and plasmid constructs used in the study. (DOCX)

S2 Table Vaccines used in the study. (DOCX)
