# Supplementary material for: Oral Delivery of a Novel Attenuated Salmonella Vaccine Expressing Influenza A Virus Proteins Protects Mice against H5N1 and H1N1 Viral Infection
Source: PLoS One. 2015 Jun 17;10(6):e0129276. doi: 10.1371/journal.pone.0129276 (PMC4471199; doi:10.1371/journal.pone.0129276)
Supplement: S1 Table — (DOCX) [file pone.0129276.s001.docx]

**S1 Table. *Salmonella* strains and plasmid constructs used in the study.**

| **Plasmids/*Salmonella*** | **Description** | **Reference/source** |
| --- | --- | --- |
| pVAX1 | cloning vector for protein expression without tag in mammalian cells | Invitrogen |
| pET-28a(+) | cloning vector for protein expression with His tag in prokaryotic cells | Novagen |
| p5HA | pVAX1 containing influenza virus(A/Viet Nam/1194R (H5N1)) HA full length sequence | This study |
| p5NA | pVAX1 containing influenza virus(A/Viet Nam/1194R (H5N1)) NA full length sequence | This study |
| pET-28a(+)-5HA | pET-28a(+) containing influenza virus(A/Viet Nam/1194R (H5N1)) HA full length sequence | This study |
| SL14028s | Wild type *Salmonella typhimurium* strain | [[33](#_ENREF_33),[36](#_ENREF_36)] |
| SL7207 | Attenuated auxotrophic *Salmonella typhimurium* *aroA* strain (a gift from Bruce Stocker of Stanford University) | [[34](#_ENREF_34)] |
| SL368 | Attenuated *Salmonella* strain that was derived from SL7207 and in addition, contains the deletion of a part of the *spiR* gene. | This study |
