# Supplementary material for: Oral Delivery of a Novel Attenuated Salmonella Vaccine Expressing Influenza A Virus Proteins Protects Mice against H5N1 and H1N1 Viral Infection
Source: PLoS One. 2015 Jun 17;10(6):e0129276. doi: 10.1371/journal.pone.0129276 (PMC4471199; doi:10.1371/journal.pone.0129276)
Supplement: S2 Table — (DOCX) [file pone.0129276.s002.docx]

**S2 Table. Vaccines used in the study**

| **Vaccine** | **Contents** |
| --- | --- |
| Sal-vector | *Salmonella* strain SL368 containing the empty vector construct pVAX1 |
| Sal-HA-NA | *Salmonella* strain SL368 containing constructs p5HA and p5NA |
| cv-H5N1 | Commercial H5N1 vaccine (purchased from Weike Inc (Harbin, China)) |
| cv-H1N1 | Commercial H1N1 vaccine (purchase from Hualan Inc (Henan, China)) |
